# The osteoporosis treatment gap in Switzerland between 1998 and 2018

**DOI:** 10.1007/s11657-022-01206-6

**Published:** 2023-01-18

**Authors:** Kurt Lippuner, Bita Yousefi Moghadam, Patrick Schwab

**Affiliations:** 1 Department of Osteoporosis, Inselspital, Bern University Hospital, University of Bern, Bern, Switzerland; 2 Swiss Federal Statistical Office, Neuchâtel, Switzerland

**Keywords:** Osteoporosis, Treatment gap, Hip fractures, FRAX, Bisphosphonates, Denosumab

## Abstract

***Summary*:**

The annual number of patients treated for osteoporosis between 1998 and 2018 in Switzerland increased until 2008 and steadily decreased thereafter. With a continuously growing population at fracture risk exceeding an intervention threshold, the treatment gap has increased and the incidence of hip fractures has stopped declining in the past decade.

***Introduction*:**

The existence of an osteoporosis treatment gap, defined as the percentage of patients at risk for osteoporotic fractures exceeding an intervention threshold but remaining untreated, is widely acknowledged. Between 1998 and 2018, new bone active substances (BAS) indicated for the treatment of osteoporosis became available. Whether and if so to what extent these new introductions have altered the treatment gap is unknown.

***Methods*:**

The annual number of patients treated with a BAS was calculated starting from single-drug unit sales. The number of patients theoretically eligible for treatment with a BAS was estimated based on four scenarios corresponding to different intervention thresholds (one based solely on a bone mineral density *T* score threshold and three FRAX-based thresholds) and the resulting annual treatment gaps were calculated.

***Results*:**

In Switzerland, the estimated number of patients on treatment with a BAS increased from 35,901 in year 1998 to 233,381 in year 2018. However, this number grew regularly since 1998, peaked in 2008, and steadily decreased thereafter, in timely coincidence with the launch of intravenous bisphosphonates and the RANKL inhibitor denosumab. When expressed in numbers of untreated persons at risk for osteoporotic fractures exceeding a given intervention threshold, the treatment gaps were of similar magnitude in 1998 (when the first BSAs just had become available) and 2018. There was a strong association, which does not imply causation, between the proportion of patients treated and hip fracture incidence.

***Conclusion*:**

In Switzerland, the osteoporosis treatment gap has increased over the past decade. The availability of new BAS has not contributed to its decrease.

**Supplementary Information:**

The online version contains supplementary material available at 10.1007/s11657-022-01206-6.

## Introduction

Osteoporosis is a crippling disease with alterations in bone quantity and quality leading to an increased risk of fragility fractures most commonly located at the hip, spine, distal radius, and/or proximal humerus, also known as major osteoporotic fractures (MOF). The operational definition of osteoporosis proposed by the World Health Organization relies on a *T* score at or below 2.5 measured by dual-energy X-ray absorptiometry (DXA) at the femoral neck [1].

In Switzerland, approximately 1 in 2 women and 1 in 5 men will sustain a fragility fracture during their remaining lifetime after age 50 [2]. While increasing in number, the incidence and even more so the age-standardized incidence of hospitalizations for hip fractures have been shown to follow a long-lasting decreasing trend between 1998 and 2018 in both men and women [3]. During these 21 years of observation, the proportion of hospitalizations for hip fractures of all hospitalizations for MOF decreased from approximately 55% in both men and women to 44% and 42% in men and women, respectively. Among the most prescribed osteoporosis drugs in Switzerland, the aminobisphosphonates alendronate and zoledronate and the RANKL inhibitor denosumab were shown to significantly reduce the risk of hip fractures in fracture endpoint trials [4–6] and their use may have contributed to the observed reduction in hip fracture incidence.

Since the launch of the first oral bisphosphonate approved for the treatment of osteoporosis in Switzerland (alendronate in 1996), an array of drug therapies aiming at reducing fracture risk in patients with osteoporosis has been made readily available to physicians and patients, allowing for multiple therapeutic options based on different mechanisms of action, efficacy and safety profiles, and administration routes and schemes. In Switzerland, approved and reimbursed osteoporosis pharmacological treatments include bisphosphonates (as daily, weekly, or monthly tablets and as quarterly or yearly intravenous infusions), denosumab as twice yearly subcutaneous injections, teriparatide as daily subcutaneous injections, and raloxifene as daily tablets. Of note, reimbursement restrictions generally based on a DXA *T* score threshold and/or the presence of one or more prevalent fractures, apply to all except alendronate (Supplemental table S1). Off-label prescriptions and prescriptions of non-reimbursed drugs are the exception rather than the rule and can be considered negligible.

In earlier publications, different groups have used different methodologies and definitions for estimating the osteoporosis treatment gap. While the common understanding is that treatment gap defines the proportion of patients who do not get treatment although eligible for therapy based on a given intervention threshold, the source databases and the inclusion criteria differed widely. Some authors estimated the treatment gap based either on national health databases and database linkage such as the National Danish Health Registries [7] or the National Medicare Database in the US [8] and others performed prospective studies such as the Austrian ICUROS [9] and the Belgian FRISBEE [10] studies. Typically, all these studies included patients who had experienced an index major osteoporotic fracture, either only women [8, 10] or both men and women [7, 9] with diverging criteria regarding age at inclusion. The gap was then estimated on these (sub-)populations with treatment generally defined as one of the bone active substances described above and generally excluding calcium, vitamin D, and estrogens, at the exception of the study by Malle et al. in which the latter were included [9]. By contrast, the SCOPE 2021 project, aiming at standardizing the approach for cross-country comparisons (EU27 + Switzerland + United Kingdom), followed a different methodology. SCOPE 2021 defined the patient population eligible for treatment as those with a 10-year probability of MOF exceeding that of a woman with a prior fragility fracture, estimated the number of patients treated based the IQVIA drug sales database (whereby calcium, vitamin D, and estrogens were not considered), and adjusted for adherence by using a point estimate derived from the Swedish Prescribed Drugs Register [11]. Thus, treatment gap studies and results should not be compared directly without the necessary caution with regard to the methodology applied.

The aim of the present analyses was to determine the number of patients treated with a pharmacologically active osteoporosis drug in Switzerland between 1998 and 2018, to evaluate the changes in treatment patterns following the introduction of new substances, to identify and quantify a potential osteoporosis treatment gap, and to explore the association between treated patients and changes in hip fracture incidence.

## Methods

Yearly sales of counting units (i.e., the smallest available single dosage of a preparation, e.g., a tablet or a vial) of all pharmacologically active osteoporosis drugs including generics in all formulations, dosage strengths, and presentations available in Switzerland since 1998 and up to year 2018, were obtained from IQVIA Switzerland (IQVIA RDS Switzerland Sàrl, Saint-Prex, Switzerland). Pharmacologically active osteoporosis drugs were defined as oral bisphosphonates (alendronate 10 mg daily or 70 mg weekly, risedronate 5 mg daily or 35 mg weekly, ibandronate 150 mg QM, including generics), intravenous bisphosphonates (ibandronate 3 mg Q3M and zoledronate 5 mg Q12M), denosumab 60 mg Q6M, raloxifene 60 mg daily, and teriparatide 20 mcg daily. Not included were abaloparatide and strontium ranelate (not available in Switzerland) and basedoxifene (available but not reimbursed). Early bisphosphonates (including etidronate, clodronate, and pamidronate) were neither available in Switzerland nor approved for the treatment of osteoporosis and thus not included in the analysis. Counting units were converted into treatment-years by applying the annualized recommended dose for the treatment of osteoporosis published in the Swiss prescribing information (https://compendium.ch). Of note, prescription data were not available by sex, such that only the total number of patients treated could be calculated.

In the absence of Swiss data, persistence defined as the duration of time from initiation to discontinuation of therapy was calculated based on previously published findings in women with US Medicare fee-for-service coverage [12]. The annual persistence rates of women with records covering three years or more were averaged leading to values of 0.596, 0.476, and 0.312 for denosumab, intravenous bisphosphonates, and oral bisphosphonates, respectively. The number of patients on treatment with a pharmacologically active osteoporosis drug in a given year was obtained by multiplying the number of treatment-days for each substance with the corresponding persistence values. It was assumed that persistence did not change between 1998 and 2018.

The total number of patients eligible for treatment (patient potential) was estimated based on four scenarios. For all, the annual Swiss population statistics by sex and 5-year age groups after age 45 published by the Swiss Federal Office of Statistics were used as a starting point. The most conservative scenario considered that the patient potential was restricted to men and women with a *T* score at or below − 2.5 at the femoral neck or the lumbar spine. The latter was estimated based on the published US osteoporosis prevalence rates of 3.9% and 15.8% in non-Hispanic White men and women [13]. In this study, reference groups used for the femoral neck and the lumbar spine were 20–29 year-old non-Hispanic White females from NHANES III and 30-year old White females from the DXA manufacturer database, respectively [13]. The three other scenarios for estimating the patient potential were defined based on FRAX risk thresholds by 5-year age groups for MOF in men and women. The FRAX algorithm allows for the country-specific calculation of the individual 10-year absolute risk of either hip fracture or MOF based on clinical risk factors with or without BMD. For the purpose of this analysis, the number of men and women aged 45 years or older with a FRAX risk for MOF exceeding 15% or 20% or exceeding the risk of a Swiss woman with a prior fracture was calculated based on previously published prevalence data used for the Swiss-specific FRAX model (https://www.sheffield.ac.uk/FRAX/) calibrated for Swiss-specific fracture risk and life expectancy [2, 14, 15]. It was assumed that fracture risk in the age group 45–49 years was identical with that in the age group 50–54 years. The thresholds of 15% and 20% were chosen because osteoporosis treatment had previously been shown to be cost-effective in the Swiss healthcare setting with a 10-year probability for a MOF at or above 15.1% (range 9.9 to 19.9%) and 13.8% (range 10.8 to 15.0%) in men and women, respectively [14]. The prior fracture risk equivalent threshold, i.e., the 10-year fracture probability for MOF exceeding that of a woman with a prior fragility fracture according to the FRAX algorithm calibrated for Switzerland, was chosen because of its general acceptance in the Swiss health economic context (almost all drugs are reimbursed in the presence of a positive fracture history) and in the latest recommendations for osteoporosis treatment from the Swiss Association Against Osteoporosis (SVGO/ASCO) [16]. Furthermore, earlier findings suggested that osteoporosis treatment was cost-effective or cost-saving in the Swiss setting after the age of 55 years in men and 60 in women who had previously sustained a fragility fracture [14, 15]. For each FRAX-based scenario, the age-specific proportion of men and women qualifying for the predefined fracture risk categories was applied to the annual structure of the Swiss population from year 1998 through year 2018.

The annual osteoporosis treatment gap was defined as the absolute or relative difference between the patient potential and the number of treated patients between 1998 and 2018.

The number of hospitalizations for hip fractures in men and women aged 45 years and older were extracted for the years 1998 through 2018 from the Swiss Federal Office of Statistics health database using the ICD-10 codes S72.0 Fracture of the femoral neck, S72.1 Pertrochanteric fracture, and S72.2 Subtrochanteric fracture) [3]. The crude incidence of hip fractures in men and women pooled was calculated per 100,000 person-years. In the absence of available covariates, the degree of association between the number of patients treated by year and the earlier reported decrease in hip fracture incidence [3] was explored by univariate regression analyses. It was considered that all hip fractures were hospitalized.

## Results

### Treatment-years with pharmacologically active osteoporosis drugs between 1998 and 2018

**Fig. 1:**
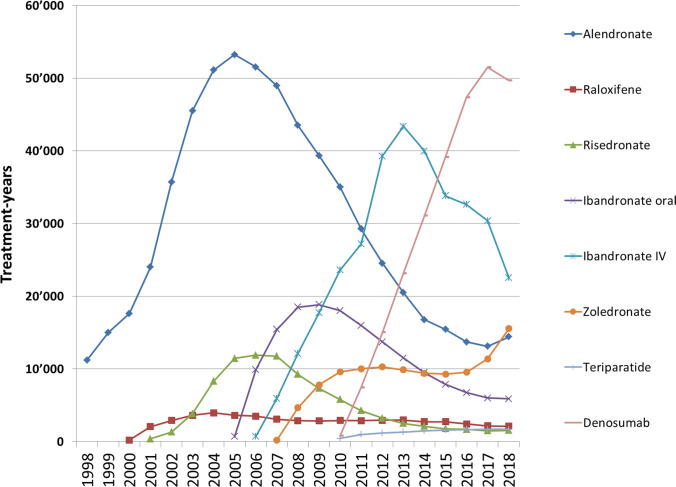
Treatment-years by pharmacologically active osteoporosis drug

Figure 1 shows the treatment-years (assuming full persistence and compliance) by pharmacologically active osteoporosis drug and year since 1998. The cumulated total number of patient-years of therapy in the IQVIA database was 1.894 Mio during 21 years of observation of which 914,979 with oral bisphosphonates (48.3%), 547,600 with IV bisphosphonates (28.9%), 359,814 with denosumab (19.0%), 56,333 with raloxifene (3.0%), and 15,430 with teriparatide (0.8%). Thus, raloxifene and teriparatide were excluded from further processing.

### Patients treated

After adjustment for persistence and as shown in Table 1, the estimated number of patients on treatment with a pharmacologically active osteoporosis drug increased from 35,901 in year 1998, i.e., two years after the launch of alendronate, to 233,381 in year 2018. While in year 1998, all patients were treated with alendronate as the only available option, in year 2018, 35.7% were treated with denosumab, 34.3% with an intravenous bisphosphonate (ibandronate 20.3%), and 30.0% with an oral bisphosphonate (alendronate 19.8%). The number of patients treated grew regularly since 1998, peaked in 2008, flattened in 2009, and steadily decreased since then. Of note, the IV bisphosphonates were launched in Switzerland in 2006 (ibandronate) and 2007 (zoledronate) and denosumab in 2010.

**Table 1:** Number of patients treated with a pharmacologically active osteoporosis drug per year

	Oral bisphosphonates			Intravenous bisphosphonates		Denosumab	Total patients
	Alendronate	Risedronate	Ibandronate	Ibandronate IV	Zoledronate		
1998	35,901						**35,901**
1999	48,076						**48,076**
2000	56,506						**56,506**
2001	76,893	1195					**78,088**
2002	114,470	4214					**118,684**
2003	145,868	12,258					**158,127**
2004	163,799	26,596					**190,395**
2005	170,610	36,649	2175				**209,434**
2006	165,127	38,132	31,736	1493			**236,489**
2007	156,833	37,675	49,503	12,488	452		**256,952**
2008	139,446	29,654	59,318	25,428	9779		**263,625**
2009	126,101	23,452	60,354	37,202	16,380		**263,489**
2010	112,210	18,600	57,757	49,558	20,176	1337	**259,638**
2011	93,771	13,697	51,147	57,106	21,021	12,469	**249,210**
2012	78,623	10,286	43,948	82,538	21,565	25,232	**262,193**
2013	65,659	8007	36,817	91,065	20,737	38,878	**261,163**
2014	53,768	6734	30,377	83,956	19,754	52,130	**246,720**
2015	49,461	5704	25,242	71,037	19,515	65,721	**236,680**
2016	43,942	5406	21,529	68,497	20,059	79,492	**238,924**
2017	41,980	4758	19,217	63,833	23,914	86,387	**240,089**
2018	46,201	4946	18,881	47,338	32,679	83,336	**233,381**

Values in bold indicate the line total

### Patient potential based on different scenarios

**Fig. 2:**
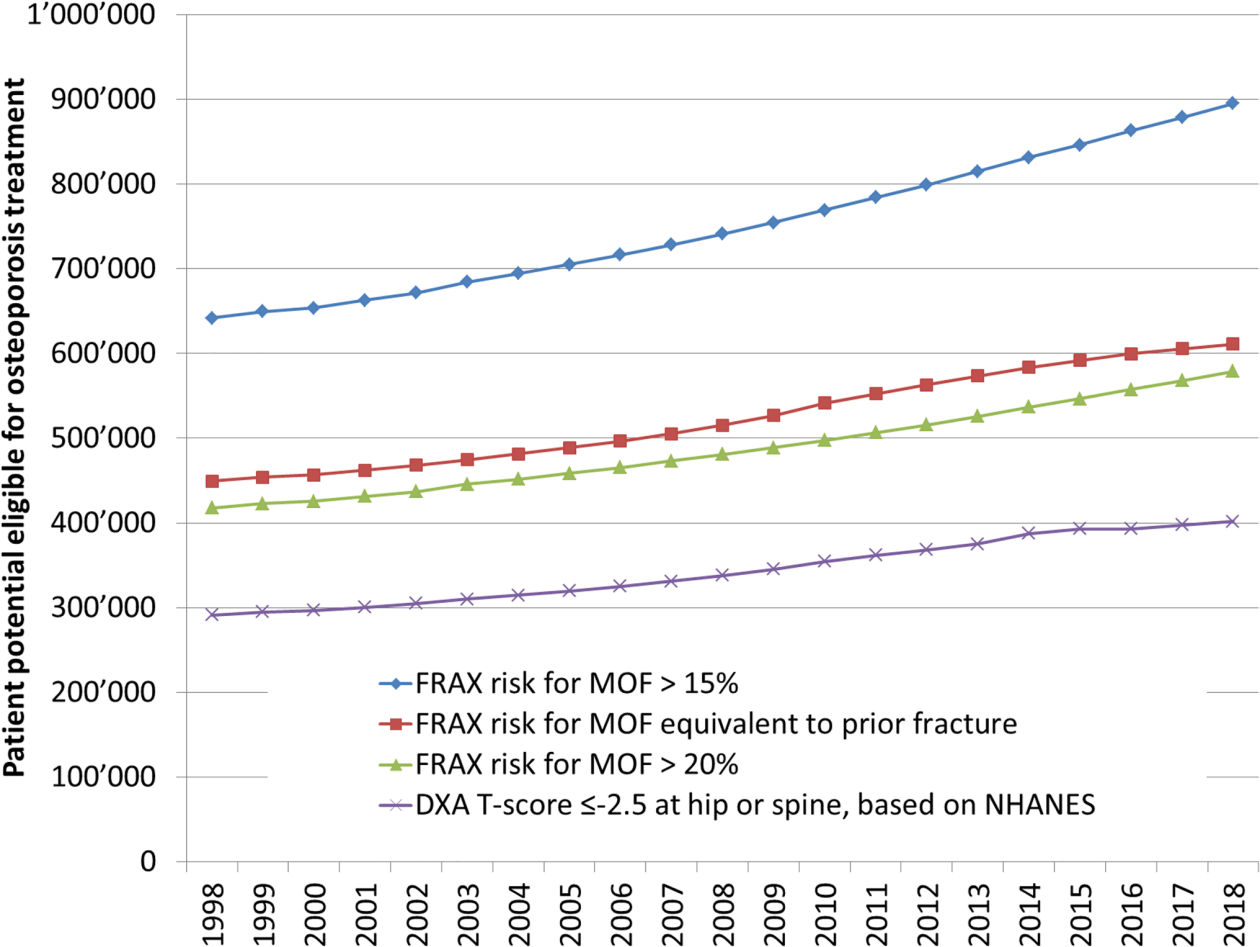
Patient potential eligible for treatment according to different risk level-based intervention thresholds for osteoporotic fractures

The DXA *T* score ≤  − 2.5 scenario based on NHANES data used the US prevalence rates of 3.9% and 15.8% in non-Hispanic White men and women, respectively. For all scenarios based on FRAX Switzerland, the proportion of patients applied to the annual population structure is shown by 5-year age groups and sex in Table 2. The aggregated numbers of patients eligible for osteoporosis treatment between 1998 and 2018 are shown by scenario in Fig. 2. In all scenarios, the patient potential increased by 36.0 to 39.5% during the 21-year observation period, mainly reflecting the fast aging of the Swiss population. When considering patients with a DXA *T* score at or below − 2.5 at either the hip or the lumbar spine, the absolute number of patients (men and women) eligible for treatment based on the DXA-based operational definition of osteoporosis increased from 291,258 to 401,751 (+ 110,493) between 1998 and 2018. At the other extreme, the number of patients with a FRAX-based risk for MOF exceeding 15%, at which it still was cost-effective to treat osteoporosis in Switzerland, increased from 641,687 to 895,310 (+ 253,623).

**Table 2:** Proportion of men and women with a FRAX risk for MOF exceeding a predefined threshold, by 5-year age groups

Age group (years)	FRAX risk for MOF > 15%		FRAX risk for MOF > 20%		FRAX risk for MOF above risk with prior fracture	
	Men (%)	Women (%)	Men (%)	Women (%)	Men (%)	Women (%)
45–49	0.9	5.4	0.2	1.6	6.4	25.8
50–54	0.9	5.4	0.2	1.6	6.4	25.8
55–59	2.1	16.0	0.6	6.5	3.7	22.8
60–64	3.3	26.3	1.0	12.3	2.7	22.7
65–69	6.0	46.6	2.2	26.6	3.1	32.9
70–74	10.8	74.9	4.9	53.1	2.5	36.3
75–79	18.5	86.5	9.0	69.6	1.1	25.2
80–84	23.8	85.8	12.7	70.1	1.1	21.8
85 +	22.8	76.1	12.4	59.9	1.0	17.5

### Treatment gap

**Fig. 3:**
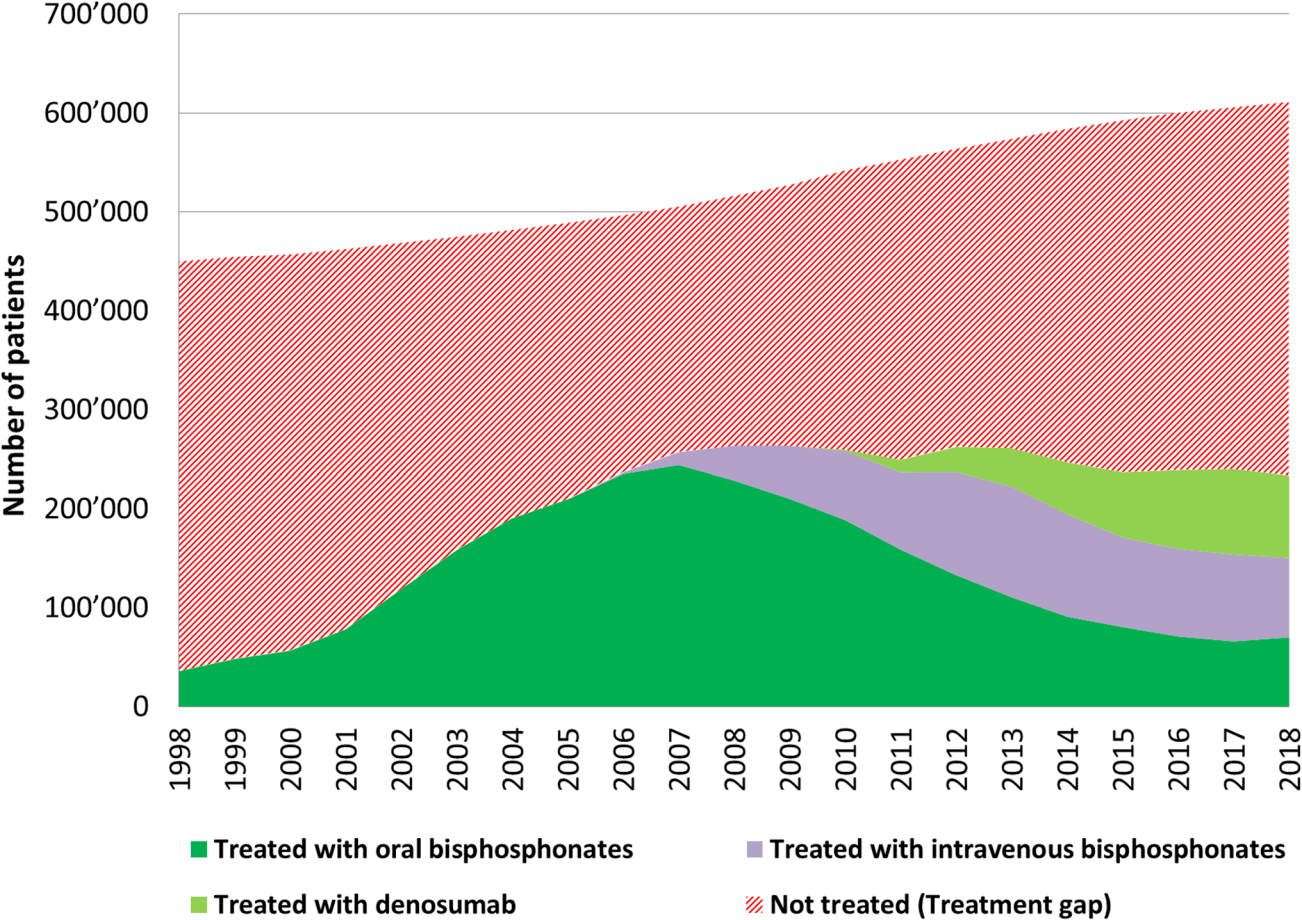
Number of patients treated and number of individuals with a 10-year fracture probability for MOF exceeding that of a woman with a prior fragility fracture based on country-specific FRAX between 1998 and 2018

The osteoporosis treatment gap, defined as the difference between the potential of patients eligible for osteoporosis treatment and the number of patients treated, varied based on the intervention thresholds retained for the different scenarios (Table 3). The common pattern was a steadily decreasing treatment gap between 1998 and 2008 followed by a one-year plateau and a continuous increase up to 2018. As an example, Fig. 3 depicts the number of patients treated with an oral bisphosphonate, an intravenous bisphosphonate, or denosumab between 1998 and 2018 and the corresponding treatment gap for patients with a 10-year fracture probability for MOF exceeding that of a woman with a prior fragility fracture according to the FRAX algorithm calibrated for Switzerland. While the number of treated patients stopped increasing and even decreased after the introduction of the intravenous bisphosphonates and denosumab, the number of patients eligible for treatment continued to increase steadily, resulting in a growing treatment gap since 2008. In this scenario, 449,328 men and women were at risk in 1998 of which 35,901 were treated leaving 413,427 patients untreated (treatment gap 92.0%). In 2018, 610,822 were at risk of which 233,381 treated and 377,501 untreated (treatment gap 61.8%). Thus, in terms of absolute numbers, the treatment gap in 2018 can be considered as only marginally different from that observed in 1998, i.e., in the very early days of availability of osteoporosis drugs proven to reduce fracture risk.

**Table 3:** Osteoporosis treatment gap between 1998 and 2018 according to different thresholds of inacceptable fracture risk justifying intervention

Year	Number of patients treated	Treatment gap if the population at inacceptable fracture risk is defined as			
		DXA *T* score ≤ − 2.5	FRAX risk for MOF > 20%	FRAX risk for MOF with prior fracture	FRAX risk for MOF > 15%
1998	35,901	87.7%	91.4%	92.0%	94.4%
1999	48,076	83.7%	88.6%	89.4%	92.6%
2000	56,506	80.9%	86.7%	87.6%	91.4%
2001	78,088	74.0%	81.9%	83.1%	88.2%
2002	118,684	61.1%	72.8%	74.6%	82.3%
2003	158,127	49.0%	64.5%	66.7%	76.9%
2004	190,395	39.5%	57.9%	60.4%	72.6%
2005	209,434	34.5%	54.3%	57.1%	70.3%
2006	236,489	27.3%	49.2%	52.4%	67.0%
2007	256,952	22.5%	45.7%	49.1%	64.7%
**2008**	**263,625**	**22.0%**	**45.2%**	**48.9%**	**64.4%**
2009	263,489	23.7%	46.1%	50.0%	65.1%
2010	259,638	26.8%	47.8%	52.1%	66.3%
2011	249,210	31.1%	50.8%	54.9%	68.2%
2012	262,193	28.8%	49.2%	53.4%	67.2%
2013	261,163	30.4%	50.4%	54.5%	67.9%
2014	246,720	36.3%	54.0%	57.7%	70.3%
2015	236,680	39.8%	56.7%	60.0%	72.0%
2016	238,924	39.2%	57.1%	60.2%	72.3%
2017	240,089	39.6%	57.7%	60.4%	72.7%
2018	233,381	41.9%	59.7%	61.8%	73.9%

Values in bold indicate the peak year for the total number of patients treated

### Association with changes in hip fracture incidence

**Fig. 4:**
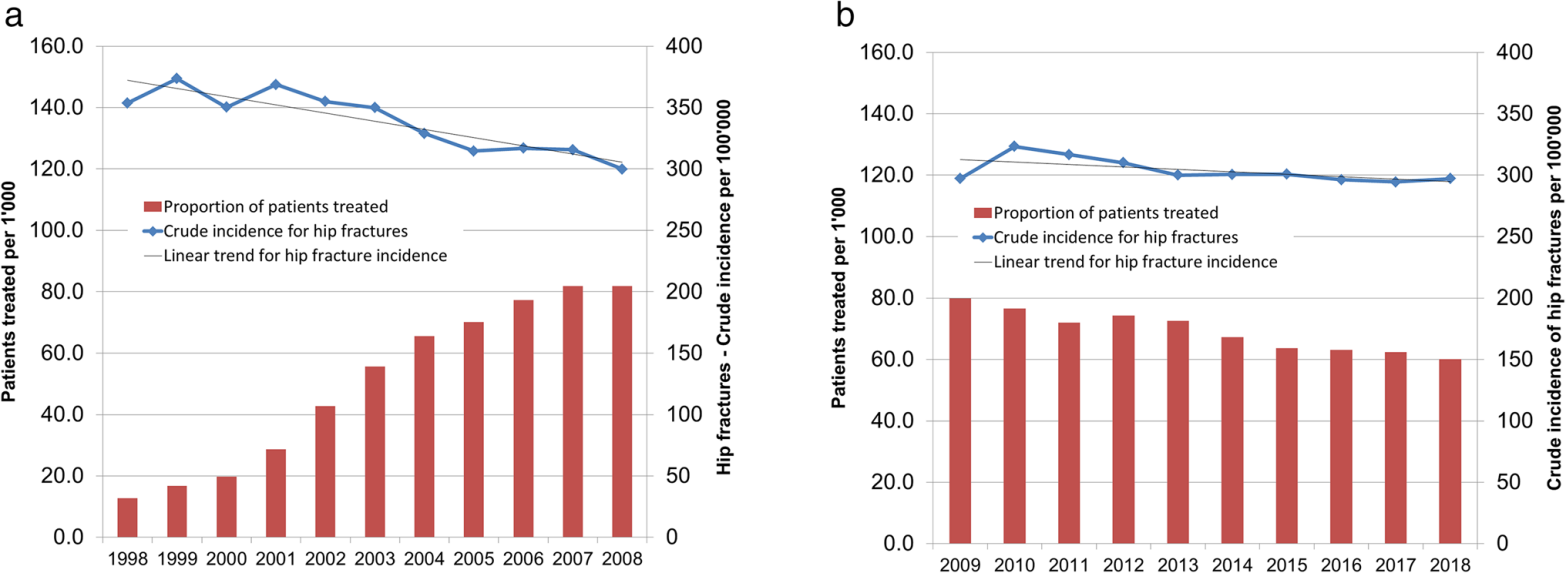
Annual changes in the crude incidence of hip fractures (line) and the proportion of patients (men and women aged 45 years and older, pooled) treated with an osteoporosis drug (bisphosphonate or denosumab) in the time periods 1998–2008 (**a**) and 2009–2018 (**b**)

In Switzerland, the number of hospitalizations for hip fractures has continuously increased since 1998 in both men and women, mainly due to the rapid aging of the population [3]. The age-standardized and, to a lesser extent, the crude incidence of hospitalizations for hip fractures have been regularly decreasing since 1998 in both men and women [3]. The crude pooled incidence of hip fractures in men and women aged 45 years or older was 354 per 100,000 persons in 1998, 300 in 2008 (Fig. 4a), 297 in 2009, and 297 in 2018 (Fig. 4b.). The corresponding proportions of patients treated for osteoporosis were 12.7, 81.9, 90.0, and 60.0 per 1000 men and women aged 45 years or older.

Between 1998 and 2018, the annual crude incidences of hip fractures in men and women aged 45 years or older (per 100,000, pooled) was not significantly associated with the proportion of these under treatment with a pharmacologically active osteoporosis drug (per 1000). However, when considering specific time intervals, this association was significant between 1998 and 2008 (*r* =  − 0.89 (95%CI − 0.97 to − 0.63), *r*^2^ = 0.80, two-sided *p* < 0.001) but not between 2009 and 2018 (*r* = 0.54 (95%CI − 0.14 to 0.87), *r*^2^ = 0.29, two-sided *p* = 0.108). Thus, the incidence of hospitalizations for hip fractures stopped decreasing together with the stagnation of and decrease in the proportion of patients treated for osteoporosis.

## Discussion

The present analysis represents a pragmatic and easily reproducible approach to the evaluation of the treatment gap. Key findings include the observation that the introduction of intravenous bisphosphonates (ibandronate and zoledronate) and of the RANKL inhibitor denosumab has not resulted in a further increase of the total number of patients treated. Of mirrored concern, the treatment gap, which had been constantly decreasing since 1998, reached its nadir in 2008 and steadily increased thereafter. Keeping in mind that the secular decrease in hip fracture incidence had already started in 1998 to 2001, i.e., at a time where the number of treated patients was low, there was a strong association, which does not imply causation, between the decrease in hip fracture incidence and the number of patients treated with a pharmacologically active osteoporosis drug.

Using a more comprehensive and therefore complex modeling approach aimed at estimating the clinical and economic burden of osteoporotic fractures in year 2010, the osteoporosis treatment gaps in men and women in Switzerland were estimated at 36 and 58%, respectively [17]. In the present analysis, the calculated treatment gap for both sexes taken together varied from 27 to 66% in year 2010 depending on the fracture risk threshold for intervention scenario considered as acceptable. Importantly, while all of these scenarios were shown to be cost-effective or even cost-sparing in the Swiss setting [14], none exactly reflects the current requirements for reimbursement of osteoporosis drugs in Switzerland. The latter stipulate *cum grano salis* that reimbursement is warranted in the presence of a *T* score at or below − 2.5 and/or a prevalent (fragility) fracture only. From a clinical perspective, treating patients having experienced one or more fragility fractures and thus all individuals with a 10-year fracture probability exceeding that of a woman with a prior fragility fracture may be more acceptable to physicians than a cost-effective intervention threshold expressed as percent risk [11, 16, 17]. However, such an approach may lead to treating a minority of patients at highest risk, while a majority of patients at lower but still increased risk may not be granted reimbursed access to treatment. In terms of total number of fractures, the end result would be that a large number of fractures would still occur in unprotected patients at risk and undermine achievable better public health results at the global population level. This triggers and further supports recent developments suggesting that a population-based screening for fracture risk in postmenopausal women based on FRAX should be considered, in addition to the currently implemented high-risk case finding and treatment approach, for incorporation in many healthcare systems to reduce the burden of fractures [18, 19].

The calculation of the number of patients on therapy should be considered with the caution relative to the underlying assumptions. The drug-specific adjustments for persistence were taken from a subset of Medicare patients with fee for service coverage who had stayed on therapy for at least three years [12]. Whether similar persistence rates apply to Switzerland is likely but not established. Whether these rates vary over time is also unknown. However, considering that persistence is generally better in patients treated with denosumab than with IV bisphosphonates and worst with oral bisphosphonates has been repeatedly shown in the literature [20–23]. More important than the absolute values, the changes over time allow for internal consistency and acceptable comparability. It is remarkable that the introduction of intravenous bisphosphonates and denosumab has not contributed to a market expansion in terms of number of patients treated, although the pool of eligible patients has increased over time due to the rapid aging of the population. A possible explanation is that at the time of launch of the early bisphosphonates all efforts were concentrated towards case finding, i.e., identifying new patients eligible for treatment aimed at reducing fracture risk within the boundaries set by reimbursement restrictions. Ten years later, when ibandronate (oral in 2005, intravenous in 2006), zoledronate (2007), and denosumab (2010) were launched, an established market of almost 250,000 patients, already identified and treated with an oral bisphosphonate, existed. In such a situation, recommended marketing strategies aim at “picking the low hanging fruits” for fast market penetration, i.e., at switching patients on therapy to the new substance. Another explanation could be that the market was already saturated, i.e., that all eligible and accessible patients had already been treated which seems unlikely in the context of a growing patient potential. Finally, the number of newly identified patients starting on an osteoporosis drug and of those already diagnosed who resume treatment after an interruption may be equal to the number of patients stopping treatment. The concept of drug holiday in low-risk patients treated during 3 to 5 years with a bisphosphonate was introduced in 2011 based on a FDA recommendation [24] triggered by considerations related to bisphosphonate accumulation in bone [25], potentially sufficient residual effects on fracture risk after treatment discontinuation in low risk patients [26–28], and safety concerns regarding rare reported of uncommon adverse events (osteonecrosis of the jaw and atypical femoral fractures) [29]. While holiday implies that treatment will be reinitiated and recommendations insisted on the importance of regular reassessments, many patients may have been lost to follow-up.

It was also intriguing to observe that the SERM raloxifene (reimbursed in Switzerland if the *T* score is at or below − 1) and the bone anabolic substance teriparatide (reimbursed as a second-line therapy after a new fracture occurring under treatment with a bisphosphonate or denosumab) were virtually inexistent in terms of patients treated, both representing less than 2% of the treatment-days in year 2018. According to *T* score-based US prevalence data, 15.8% of all White non-Hispanic women should have a *T* score at or below − 2.5 and 52.6% a *T* score at or below − 1 which corresponds to more than a tripling of the patient potential. Obviously, osteoporosis treatment is reserved to those at highest risk and little to no pharmacological intervention is the rule in patients with osteopenia. By contrast and consistent with the available published evidence, many patients with highest fracture risk (e.g., patients with multiple vertebral fractures) should have been eligible for treatment with the only bone anabolic substance available during the period of observation under scrutiny, namely, teriparatide [30]. Here again, available data suggest important underuse. Overall, these findings plead in favor of an urgent need for drug prescription data with higher granularity which would allow evaluating the treatment gap by risk categories and by sex.

Overall, the present findings suggest that the key challenges for overcoming the treatment gap published in a 2017 narrative global perspective on strategies for the prevention of fragility fractures need more urgent attention than ever [31]. Case finding and management of individuals at high risk of fracture should be revitalized, raising public awareness about osteoporosis and fragility fractures should be amplified or reinitiated, improving reimbursement and health system policies should ensure access to the most appropriate treatments to those patients expected to benefit most, and optimizing our epidemiological understanding of osteoporosis and fractures should include tools for regular progress assessment. For that, the newly proposed scorecard within the SCOPE project should be considered as an excellent starting point to be tailored to individual countries’ specific needs [11].

Among the strengths of this study is the straightforward and reproducible approach to the treatment gap calculation ensuring internal data consistency and allowing for monitoring changes over time. It also overcomes the challenges met when trying to estimate treated patient numbers based on DDDs (defined daily dosages). The latter are mandated by the WHO in order to ensure worldwide data comparability but sometimes suffer from important deviations when put into perspective with the approved dosing schemes. Limitations include that the IQVIA drug sales database does not report drug use by indication. While some of the drugs used for the treatment of osteoporosis have additional indications (such as the prevention of bone loss under antihormonal treatments or Paget’s disease of bone), the impact on the number treated patients can be considered as limited. The latter is further warranted by the existence of specific formulations for additional indications unrelated to osteoporosis, such as zoledronate 4 mg or denosumab 120 mg monthly, both for oncological indications, which were excluded from the present analysis. The IQVIA database does not report drug use by sex, such that an overall approach pooling men and women was chosen at the expense of a loss in data granularity. The IQVIA database does not either collect clinical characteristics which also preempted a more detailed analysis by gradients of fracture risk. Assumptions regarding persistence and DXA-based osteoporosis prevalence where taken from US publications which may differ from the Swiss reality, highlighting the urgent need for further epidemiological research at the individual country level. In the absence of better data, it was assumed that persistence, which addresses the question of how long a patient stays on therapy after treatment initiation, did not change between 1998 and 2018. Whether and to what extent prescription behavior and persistence have been impacted by real or suspected emerging safety concerns with antiresorptives, including the scientific discussions around atypical femoral fractures, osteonecrosis of the jaw, and atrial fibrillation events, remains unknown and deserves further research. On the other hand and complementary to that, adherence to the prescription, i.e., the extent to which a patient acts in accordance with the prescribed interval and dose of a dosing regimen, would be important to know and adjust for, especially for oral drugs. Both adherence and persistence have been shown important for optimizing fracture outcomes [32, 33]. Finally, we did not retain hormone replacement therapy (HRT) in our selection of bone active substances, albeit it was shown to significantly reduce hip fracture risk by approximately one-third in the Women’s Health Initiative (WHI) trials [34, 35]. The WHI trials also showed a significant increase in breast cancers, cardiovascular diseases, and all-cause mortality which led to drastic reductions in systemic HRT use since 2002, now being mainly reserved to younger postmenopausal women with vasomotor symptoms [36, 37]. As the effect of HRT on hip fracture risk was shown having vanished three years after discontinuation [38], we consider that omitting HRT from our analyses should not be expected to alter the present observations and conclusions. Whether and to what extent changes in the HRT prescription patterns due to safety concerns that emerged during the observation period covered by the present analyses may have contributed to the observed slowing of the secular decrease in hip fracture incidence deserves further research.

Overall, the present study suggests that although multiple treatment options were made available over the past two decades, the treatment gap remains large and has even increased over the past decade. Case finding strategies which may have been neglected in the more recent past should be reassessed. Future research should focus on establishing instruments for measuring progresses made, including on more detailed datasets reporting drug usage by sex and indication.

### Supplementary Information

Below is the link to the electronic supplementary material.

Supplementary file1 (DOC 40 KB)

## Data Availability

Source data are available from the corresponding author upon request.
