# Supplementary material for: The osteoporosis treatment gap in Switzerland between 1998 and 2018
Source: Arch Osteoporos. 2023 Jan 18;18(1):20. doi: 10.1007/s11657-022-01206-6 (PMC9845158; doi:10.1007/s11657-022-01206-6)
Supplement: Supplementary file 1 — Supplementary file1 (DOC 40 KB) [file 11657_2022_1206_MOESM1_ESM.doc]

**Supplemental Table S1 – Synoptic view of applicable reimbursement restrictions for osteoporosis drugs in Switzerland. Full wording available in German, French and Italian at http://www.spezialitätenliste.ch/**

| **Substance** | **Reimbursement restrictions** |
| --- | --- |
| Alendronate | None. |
| Risedronate | T-score ≤ -2 or fracture in men and women.  Paget’s disease of bone. |
| Ibandronate | T-score ≤ -2.5 or fracture in men and women. |
| Zoledronate | T-score ≤ -2.5 or fracture in men and women, including glucocorticoid-induced osteoporosis.  Paget’s disease of bone. |
| Denosumab | T-score ≤ -2.5 or fracture in men and women.  Breast cancer during treatment with aromatase inhibitors.  Prostate cancer during antihormonal treatment. |
| Teriparatide | Second line in patients with new vertebral fractures under treatment with a SERM, denosumab or a bisphosphonate.  Second line in patients with glucocorticoid-induced osteoporosis if a bisphosphonate is ineffective or not tolerated. |
| Raloxifene | T-score ≤ -1 or fracture. |
